# Fe^III^ Chelated with Humic Acid with Easy Synthesis Conditions and Good Performance as Anode Materials for Lithium-Ion Batteries

**DOI:** 10.3390/ma16196477

**Published:** 2023-09-29

**Authors:** Hao Zhang, Youkui Wang, Ruili Zhao, Meimei Kou, Mengyao Guo, Ke Xu, Gang Tian, Xinting Wei, Song Jiang, Qing Yuan, Jinsheng Zhao

**Affiliations:** 1 School of Chemistry and Chemical Engineering, Liaocheng University, Liaocheng 252059, China; zh1836958@163.com (H.Z.); m19861904240@163.com (Y.W.); zhao1784308@163.com (R.Z.); kmm2018705344@163.com (M.K.); gmy15275641513@163.com (M.G.); x15866598163@163.com (K.X.); jiangsong006@163.com (S.J.); 2 Shandong Tianyi New Energy Co., Ltd., Liaocheng 252059, China; tg1999@163.com (G.T.); 18863588007@139.com (X.W.); 3 Shandong Provincial Key Laboratory of Chemical Energy Storage and Novel Cell Technology, Liaocheng University, Liaocheng 252059, China

**Keywords:** lithium-ion battery, anode material, organic anode, metal–organic compound, humic acid, Fe chelate

## Abstract

In this work, we prepared a green, cheap material by chelating humic acid with ferric ions (HA-Fe) and used it as an anode material in LIBs for the first time. From the SEM, TEM, XPS, XRD, and nitrogen adsorption–desorption experimental results, it was found that the ferric ion can chelate with humic acid successfully under mild conditions and can increase the surface area of materials. Taking advantage of the chelation between the ferric ions and HA, the capacity of HA-Fe is 586 mAh·g^−1^ at 0.1 A·g^−1^ after 1000 cycles. Moreover, benefitting from the chelation effect, the activation degree of HA-Fe (about 8 times) is seriously improved compared with pure HA material (about 2 times) during the change–discharge process. The capacity retention ratio of HA-Fe is 55.63% when the current density increased from 0.05 A·g^−1^ to 1 A·g^−1^, which is higher than that of HA (32.55%) and Fe (24.85%). In the end, the storage mechanism of HA-Fe was investigated with ex-situ XPS measurements, and it was found that the C=O and C=C bonds are the activation sites for storage Li ions but have different redox voltages.

## 1. Introduction

Rechargeable LIBs have attracted a great deal of attention due to their high energy density and good cycle stability as a new type of energy storage device [1,2,3,4,5,6]. LIBs are used in many applications, including portable electronics, electric vehicles, energy storage devices, and many others [7,8,9,10,11,12]. Anode material is a critical part of high-power LIBs. At present, due to poor rate performance or low theoretical capacity, inorganic materials struggle to meet the demands of high-performance batteries [13]. For example, graphite carbon materials have been used as anode materials for commercial lithium-ion batteries due to their good cycle stability and accessibility. However, further use in high-performance LIBs is hindered by the low theoretical capacity (373 mAh·g^−1^) and poor rate performance [14,15]. Silicon materials have a serious volume effect in the repeated charge and discharge process, resulting in a rapid capacity fading and a poor cycle stability, which is also a problem to be overcome before the commercial application of LIBs [16,17]. In addition, most inorganic material production processes are based on the redox reaction of inorganic compounds, containing expensive transition metal elements [18,19,20].

Organic electrode materials, due to their low cost, light weight, and abundance in nature, are considered as substitute materials to overcome the disadvantages of inorganic materials [21,22,23,24,25,26]. Until now, electrode materials for LIBs have consisted of many organic materials, including organosulfur materials [27,28,29,30], free radical compounds [31,32,33,34], conducting polymers [35,36,37], aromatic amines [38], quinine-type carbonyl compounds [39,40], and other organic materials [41]. Among them, the metal–organic polymer is an important material due to the π-d conjugated coordination. Using tetraaminobenzoquinone (TABQ) as the organic ligand and M^2+^ (M=Co, Ni, and Cu) as the metal ligands, Li et al. developed the metal–organic polymer as a cathode material of lithium-ion batteries with good rate performance (even 237.2 mAh·g^−1^ at 2 A·g^−1^) [42]. Wu et al. reported two Ni-based conjugated coordination polymers with N and S as co-chelating atoms, and it was found that the co-existence of N and S resulted in high electrical conductivity and high stability, leading to a high capacity, excellent cyclic performance, and a high rate of capability [43]. Yang et al. reported a highly stable Zn, Ni-bimetallic porous nanocomposite via a one-step pyrolysis of a metal–organic framework as an efficient anode material (1105.2 mAh·g^−1^ at 0.5 A·g^−1^ after 400 cycles) [44]. However, the practical application of organic electrodes in lithium-ion batteries has been hampered by the fact that the majority of reported organics are derived from chemical feedstock, which are expensive and environmentally problematic [45,46,47,48]. Therefore, it is very urgent to develop new electrode materials for LIBs with higher energy density, environmental friendliness, low cost, good rate performance, and long cycle life [49]. Developing new materials originating from natural sources is an important way to exploit new green, economic anode. Zhang et al. generated a novel anode material by chelating the tannic acid via ferric ions (TA-Fe) and used the anode materials with high reversible capacity (1105.2 mAh·g^−1^ at 0.1 A·g^−1^) and ultra-long cycling stability (10.0 A·g^−1^ over 16,000 cycles with a capacity retention of 78.8%) [50] Zhang et al. chelated the rhodizonic acid disodium salt (RA) using ferric ions and generated a novel organic anode material (RAFe) for the first time. The strong chelation interaction between ferric ions and rhodizonic acid changes its initial structure and characteristics, enabling the obtained organic RAFe compound with outstanding electrochemical performance as an anode for lithium-ion batteries (LIB) (1283 mAh·g^−1^ at 0.1 A·g^−1^) [51]. Zhu et al. used the humic acid as anode material for lithium-ion batteries and sodium ion batteries because of its richness in oxygen functional groups (carboxylic, quinonic, phenolic, and ketonic). However, the capability and rate performance of HA at higher currents are not excellent. The reported specific capacity of HA is 420 mAh·g^−1^ at 20 mA·g^−1^, while it is only 60 mAh·g^−1^ at 400 mA·g^−1^, whose capacity retention is 14.29% [45]. In addition, the long cycle performance is also worse than that of the traditionally reported organic materials at the current density of 40 mA·g^−1^. Inspired by the promotion method of TA-Fe anode material and the RA-Fe using ferric ions, a Fe^3+^ chelated humic acid (named as HA-Fe) in this work at easy synthesis conditions is developed as the anode material for Li-ion batteries. Unlike previously reported organic compounds or metal oxides as anodes, which frequently involve a large number of complex synthetic processes, high temperatures, and toxic pollutant emissions [50], the HA-Fe in this work can be obtained under easy conditions without high energy consumption and has no environmental impact, which is beneficial for large-scale production. From the experimental results, it can be seen that the ferric acid can increase the surface area of the HA-Fe and improve the properties of capacity (586 mAh·g^−1^ at 0.1 A·g^−1^), cycle stability (1000 cycles), and good rate properties (capacity retention ratio of 55.63%).

## 2. Materials and Methods

### 2.1. Materials

**Figure 1:**
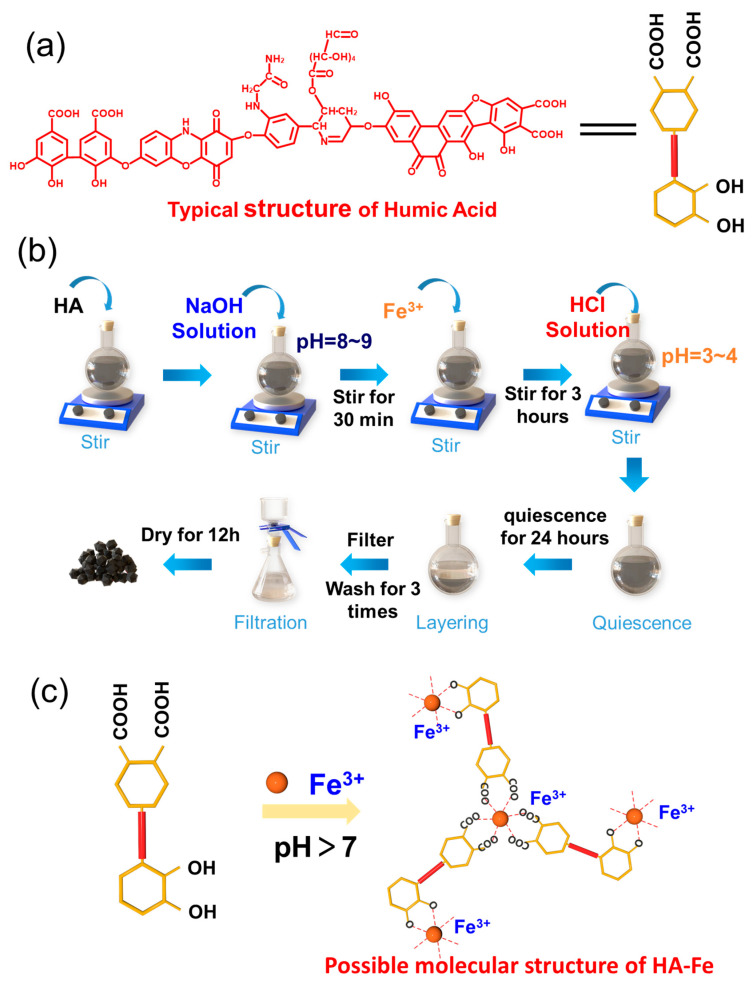
(**a**) The typical structure of HA molecules. (**b**) The formation procedures program of HA-Fe material. (**c**) Possible molecular structure of HA-Fe compound.

Humic acid (HA) was purchased from Alfa Aesar Chemical Co., Ltd., Shanghai, China (Purity, 99%), and the typical structure is shown in Figure 1a. Ammonium iron (III) sulfate (NH_4_Fe(SO_4_)_2_) was purchased from Damas-beta Chemical Co., Ltd., Shanghai, China (Purity, 99%). Hydrochloric acid (HCl) (Purity, AR) and sodium hydroxide (NaOH) (Purity, AR) were purchased from Yantai Yuandong Fine Chem. Co., Ltd., Yantai, China. All of the above chemicals were used as received without further processing. The deionized water was made in our laboratory.

### 2.2. Preparation Procedure of HA-Fe Materials

The experimental set-up is composed of a single mouth bottom flask, a magnetic stirrer, vacuum filter, and a vacuum oven. Figure 1b shows the simple preparation process of HA-Fe. 375 mg of HA was accurately weighed and dispersed in 250 mL deionized water in a 500 mL single mouth bottom flask with continuous stirring by a magnetic stirrer. Following that, 0.1 mol·L^−1^ NaOH solution was used to adjust the pH of the suspension to 8~9 to help dissolve the HA. The solution was stirred for 30 min, and then 1000 mg of NH_4_Fe(SO_4_)_2_ was added into the HA solution. The ferric ions were successfully chelated with HA after continuously stirring the mixture for 3 h at room temperature. Adjusting the pH to 3~4 with 0.1 mol·L^−1^ HCl solution, and then a large amount of black powder was precipitated from the solution. Stirring was stopped, and the mixture kept quiescence for 24 h to layer the HA-Fe material. The supernatant liquid was removed, and the underling materials were filtered and washed with deionized water for three times with a vacuum filter. The black HA-Fe powder was obtained after drying at 80 °C for 12 h in the vacuum oven. Figure 1c shows the chelation mechanism diagram and the possible molecular structure of HA-Fe.

### 2.3. Characterization and Electrochemical Properties of Materials

The Supporting Information (SI) provides the test methods and associated instrumentation information for structure confirmation, morphological characterization, and other physical characterization. The SI also provides a comprehensive introduction of the electrode preparation procedures, electrochemical testing methods, and instrumentation.

## 3. Results and Discussion

### 3.1. Characterization Results of HA and HA-Fe Materials

SEM has been used for microstructural and morphological characterization of HA-Fe and HA. As clearly depicted in Figure S1a, the original HA shows large particles, about 5–15 um in size. Interestingly, after chelating with ferric ions, the particles of HA-Fe were much smaller, as shown in Figure S1c. The smaller particle size can always reduce the lithium-ion diffusion distance, which often results in high rates of capability and good electrochemical performance [52]. From the morphology comparison in Figure S1b,d, it can be seen that the surface morphology of HA (Figure S1b) is much clearer and tighter than that of HA-Fe (Figure S1d), which illustrated that after chelating with the ferric ion, the structure of particles was changed to some extent. The homogeneous elemental distribution of oxygen and iron elements in HA-Fe materials is illustrated by the corresponding elemental mapping images in Figure S1f,g.

**Figure 2:**
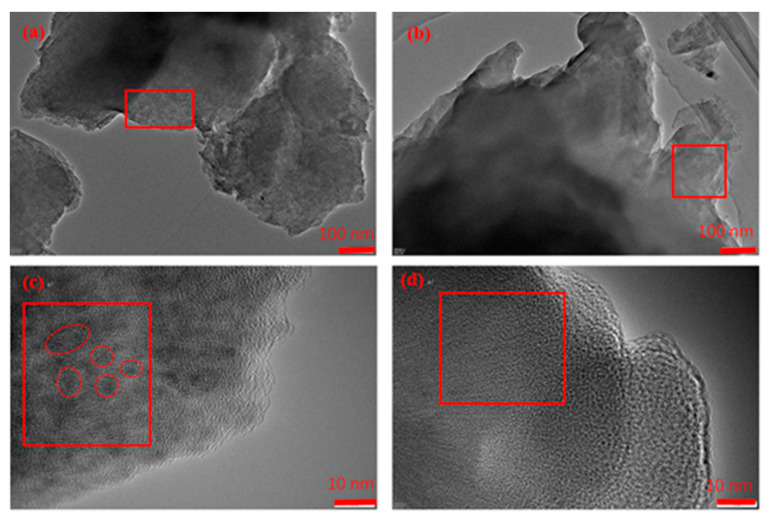
(**a**,**c**) The TEM of HA-Fe at the dimension of 100 nm and 10 nm, respectively; (**b**,**d**) The TEM of HA at the dimension of 100 nm and 10 nm, respectively.

The TEM of HA-Fe at the dimension of 100 nm and 10 nm are shown in Figure 2a and c, respectively, while the TEM of HA at the dimension of 100 nm and 10 nm are shown in Figure 2b and d, respectively. It can be seen from the areas enclosed by the red boxes in Figure 2a,b that the morphology of HA-Fe and HA were very different. There are many dark pots uniformly distributed in the materials enclosed by the red box in Figure 2a, however, the morphology of HA in the area enclosed by the red box in Figure 2b is very clear. Similarly, the blocky morphology of HA-Fe in Figure 2c, which is enclosed by the red cycles, is very different to the uniform morphology of HA enclosed by the red box in Figure 2d at the 10 nm dimension, as shown in Figure 2c,d. The different morphology illustrated that the ferric ion is chelated with the HA successfully. The mass fractions of C, O, and Fe on the surface spectrum of HA and HA-Fe are shown in Table 1. It can be seen that the mass fraction of Fe increased from <0.1% to 29.2%, which also illustrated the successful formation of HA-Fe.

**Figure 3:**
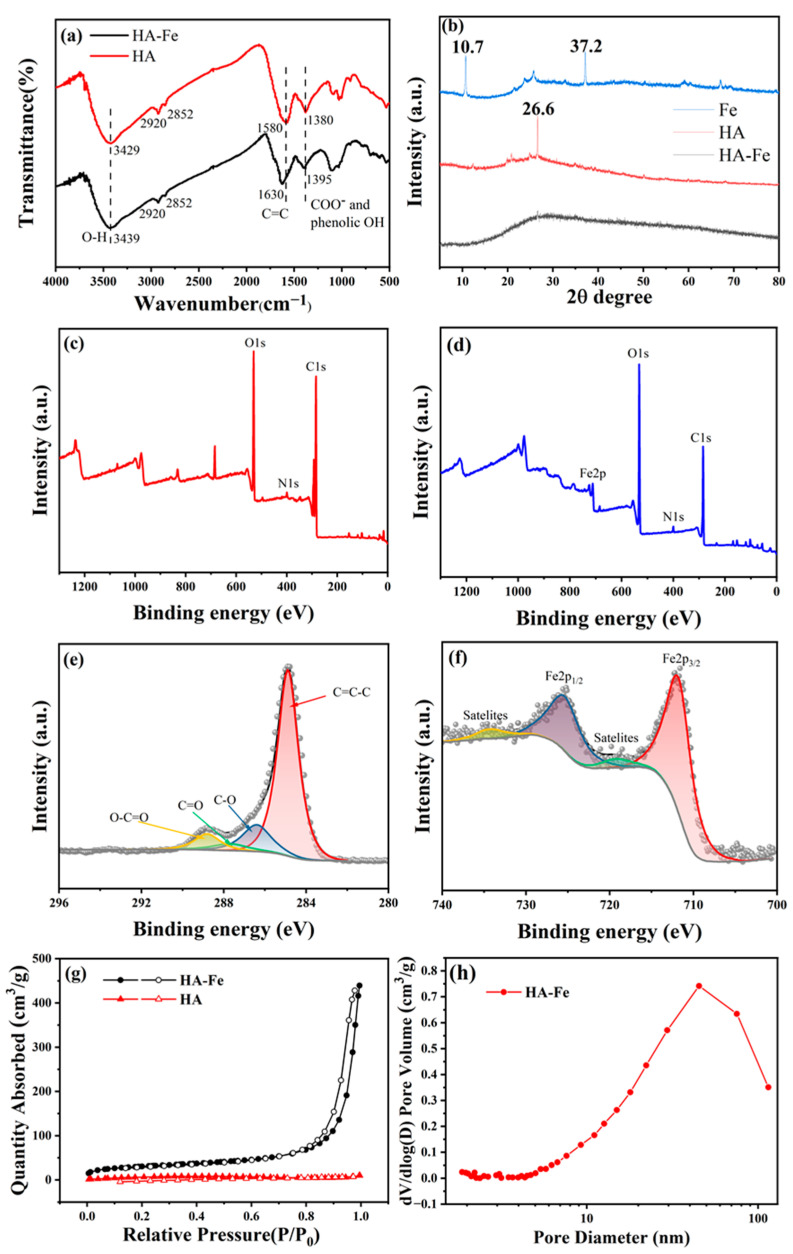
(**a**) FTIR spectra of Fe, HA, and HA-Fe. (**b**) X-ray diffraction pattern of Fe, HA, and HA-Fe. (**c**,**d**) X-ray photoelectron spectroscopy of HA and HA-Fe. (**e**,**f**) C1s and Fe2p XPS spectra of HA-Fe. (**g**) Nitrogen adsorption–desorption isotherm of HA-Fe and HA. (**h**) Pore size distribution of HA-Fe.

The FTIR spectroscopy (Thermo Fisher, USA) has been used to characterize the typical chemical groups such as carboxyl, hydroxyl groups of HA and HA-Fe. As shown in Figure 3a, the spectroscopy of HA is similar to that reported in the literature [45,53]. Also, it can be seen from Figure 3a that the infrared spectra of HA-Fe and HA are roughly the same, indicating that the structure of HA was not changed obviously after chelating with ferric acid. Specifically, the broad peak around 3429 cm^−1^ is due to the stretching of the O-H bond. The aliphatic C-H stretching of the alkyl groups and the methyl C-H groups can be assigned to the weak shoulders at 2920 cm^−1^ and 2852 cm^−1^. The peaks at 1580 cm^−1^ can be indexed to aromatic C=C, and 1380 cm^−1^ can be indexed to the symmetric stretching of COO^−^, C-OH stretching of phenolic OH [53]. However, by observing the infrared spectrum of HA-Fe carefully, the vibration peak positions of phenolic hydroxyl, aromatic C=C and COO^−^ changed slightly after forming a stable chelating structure with Fe^3+^. The O-H was transferred from 3429 cm^−1^ to 3439 cm^−1^, the aromatic C=C was transferred from 1580 cm^−1^ to 1630 cm^−1^, and the symmetric stretching of COO-, C-OH stretching of phenolic OH was transferred from 1380 cm^−1^ to 1395 cm^−1^. Similar peak shifts at the sites of OH, C=C, and COO^−^ were also observed in the spectra of Fe(III)-humic acid complex in the literature [53]. This change in the FTIR spectrum is an indication that the major functional groups chelated by the iron ions are aromatic COOH and hydroxyl or phenolic OH [53]. As is displayed in Figure 3b, XRD was used to characterize the structure of HA, Fe, and the HA-Fe materials. The strong peaks at 10.7° and 37.2° in the XRD patterns of Fe and the strong peaks at 26.6° in the XRD patterns of HA illustrated their highly crystalline nature. In comparison, no significant peaks appeared in the XRD pattern of HA-Fe after chelation with trivalent iron ions, indicating that the synthesized HA-Fe is amorphous [54].

To further confirm the chemical compositions of HA and HA-Fe, the XPS measurements of them are shown in Figure 3c–f. As shown in Figure 3c,d, two typical peaks with binding energies (BE) of 285 eV and 531 eV are assigned to the C1s and O1s orbital, respectively, indicating that the main components are carbon and oxygen [45], and the peaks at the BE of 400 eV are easily indexed to the N1s orbital, indicating that both HA and HA-Fe contain N elements [55]. By carefully observing the XPS spectrum of HA-Fe (Figure 3d), it can be seen that the newly emerging peak at the binding energy of 712 eV is indexed to the Fe2p orbital [56], indicating that Fe^3+^ has been successfully chelated to the HA molecule. The C1s spectrum of HA-Fe (Figure 3e) is divided into four different types of carbon species: C=C-C (284.6 eV), C-O (286.4 eV), C=O (287.7 eV), and O-C=O (288.7 eV) groups, which is nearly the same as the XPS spectrum of HA in the literature [45], indicating that Fe ions do not change the chemical composition significantly. Figure 3f shows the XPS spectra of the Fe element in the prepared HA-Fe sample. In the Fe2p XPS spectrum of the HA-Fe material, the two peaks at 712 eV and 725.8 eV are attributed to Fe2p_1/2_ and Fe2p_3/2_, respectively. The weak peaks at 719.3 eV and 734.7 eV are attributed to the satellite peak [57], which can also prove the successful binding of Fe^3+^.

The N_2_ adsorption and desorption at 77 K were used to study the porous structure. As shown in Figure 3g, the isotherms are assigned to the V type. Under these circumstances, the adsorption process initially resembles that of macroporous solids, and the capillaries in the mesopores will be condensed at relatively high pressure, resulting in a sharp increase in adsorption capacity. The adsorption isotherm tends to be stable after these pores have been filled. The condensation of the capillaries and the evaporation of the capillaries usually do not take place at the same pressure, which will lead to the formation of a hysteresis loop [58]. The pore size distribution curve in Figure 3h shows that the pores with various diameters (mainly distributed from 10 nm to 100 nm) are present in HA-Fe. The specific surface areas for HA-Fe and HA are 105.42 m^2^·g^−1^ and 5.01 m^2^·g^−1^, respectively, which illustrates that the ferric ion increased the surface area of HA-Fe materials. The thermal stability of HA and HA-Fe is investigated by the TGA measurement. As shown in Figure S2, the TGA curve shows that HA-Fe has better thermal stability than HA.

### 3.2. Electrochemical Properties Investigation of HA-Fe Material

In order to illustrate the electrochemical performance of the HA-Fe material as an anode for LIBs, the change–discharge process, the cycle stability, rate capability, CV curves, and electrochemical impedance were investigated systemically.

First, the CV tests were performed to study the redox reaction during charge and discharge. As shown in Figure S3a,b, the CV curves of the HA and HA-Fe materials were measured at the scanning rate of 0.1 mV·s^−1^, and the potential window is from 5 mV to 3 V (vs. Li/Li^+^). It can be seen from Figure S3a,b that the reduction current of the first charge–discharge cycle is much larger than that of the second and third cycle, which is because of the formation of SEI film [53]. As shown in Figure S3a, HA exhibited a reduction peak centered at 1.0V during the first discharge, indicating the accumulation process of lithium ions in HA materials. Meanwhile, a slightly corresponding anodic peak at 1.05 V (vs. Li/Li^+^) was detected, indicating the de-intercalation of lithium ions. Similarly, Figure S3b shows a broad oxidation peak at about 1.1 V in the first cycle, which was related to the SEI film formation and the oxidation of Fe^0^ to Fe^2+/3+^ [59]. In the subsequent cycles, the reduction peaks and the oxidation peak at 1.1 V both decreased rapidly, which is caused by the end of SEI film formation. In order to compare the response currents of HA and HA-Fe during the same scan rate clearly, the CV plot of HA and HA-Fe at the third cycle is shown in Figure S3c. It can be seen that the response current of HA-Fe is much larger than that of HA, which illustrates that the chelated ferric ion can increase the capacity greatly. Figure S3d showed the CV curves of HA-Fe materials at 3rd and 800th, respectively. It can be seen that the response current and the area enclosed by the current curve increased obviously after 800 times change–discharge process, which means that the change–discharge process can activate the electrochemical process.

**Figure 4:**
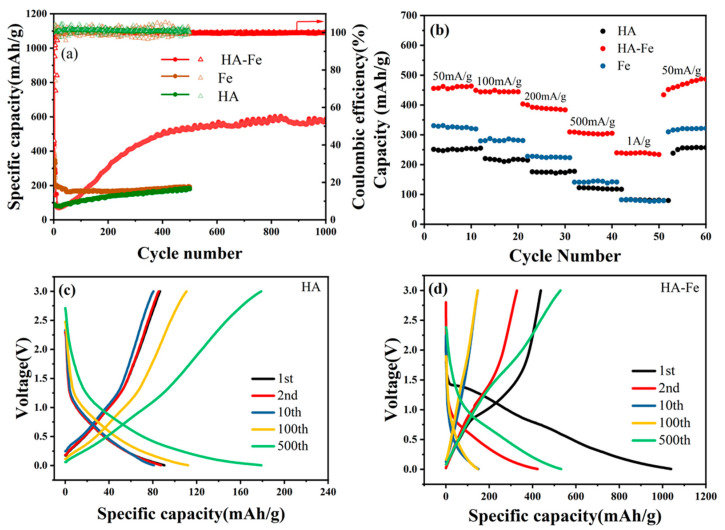
(**a**) Cycle performance diagram of Fe, HA, and HA-Fe at 100mA·g^−1^. (**b**) Rate performance diagram of Fe, HA, and HA-Fe at different current densities. (**c**,**d**) GDC curves of HA and HA-Fe at a current density of 0.1 A·g^−1^, respectively.

In order to better understand the electrochemical properties of HA-Fe materials, we tested the cycle performance of HA-Fe, HA, and ammonium iron (III) sulfate (marked as Fe). The change–discharge voltage range is 5 mV~3.0 V with a constant current of 100 mA·g^−1^. Figure 4a shows the capacity of the HA, Fe, and HA-Fe at a current density of 0.1 A·g^−1^. The initial discharge capacities of HA and Fe are about 90 mAh·g^−1^ and 385 mAh·g^−1^, respectively, which are much smaller than that of HA-Fe (1038 mAh·g^−1^). As a result, due to the increased impedance caused by the gradual formation of the SEI layer, the capacity of the three materials declines rapidly in the first few cycles. After that, the change–discharge capacities of HA and HA-Fe increased gradually to about 178 mAh·g^−1^ and 530 mAh·g^−1^ after 500 cycles, respectively. The capacity of the HA-Fe gradually leveled off after 500 cycles and stabilized at a value of 586 mAh·g^−1^ after 1000 cycles. The improvement in performance during the change–discharge cycle may be due to the activation process, e.g., the swelling process resulting from the increase in wettability of the polymer in the electrolyte, which allows for more active sites to participate in the battery’s change–discharge cycle [60]. Moreover, it can be seen that the activation degree of HA-Fe is more significant than that of HA, which may be attributed to three reasons. Firstly, as shown in the SEM morphology of HA and HA-Fe, the HA-Fe particle is much smaller and looser than that of HA. The loose character is beneficial to the lithium shuttle in the solid HA-Fe material, which makes the reaction efficiency of the lithium ions and the functional groups much higher. Secondly, from the XRD results of HA and HA-Fe, the crystallinity degree of HA materials decreased obviously after chelating with the ferric ions. Thirdly, the chelation between ferric ions and HA can dramatically decrease the solubility in electrolytes. In comparison, the change–discharge capacity of Fe did not increase during the cycling process, which means that Fe did not occur in the activation process.

In order to research the rate ability of Fe, HA, and HA-Fe, the capabilities measurements were conducted at the current densities of 0.05 A·g^−1^, 0.1 A·g^−1^, 0.2 A·g^−1^, 0.5 A·g^−1^, and 1.0 A·g^−1^, respectively, and the corresponding data are shown in Figure 4b. The HA-Fe material delivers stable charge/discharge capacities of 462 mAh·g^−1^, 447 mAh·g^−1^, 398 mAh·g^−1^, 309 mAh·g^−1^, and 252 mAh·g^−1^ at the current densities of 0.05 A·g^−1^, 0.1 A·g^−1^, 0.2 A·g^−1^, 0.5 A·g^−1^, and 1.0 A·g^−1^, respectively, while the HA material delivers stable charge/discharge capacities of 252 mAh·g^−1^, 210 mAh·g^−1^, 175 mAh·g^−1^, 120 mAh·g^−1^, and 82 mAh·g^−1^. It can be seen that at different current densities, the capacities of HA-Fe were all much higher than those of HA and Fe. The capacity retention rates of HA-Fe, HA, and Fe at 1 A·g^−1^ are 55.63%, 32.55%, and 24.85%, respectively, and the results show that the performance of the rate is significantly improved after chelation with ferric ions. In addition, the HA-Fe anode shows a 100% recovery of capacity when the current densities are returned to 0.05 A·g^−1^, which shows that the high current has not destroyed the molecular structure of the HA-Fe.

Figure 4c,d show the change–discharge curve of HA and HA-Fe, respectively. It can be seen that the main discharge potential of HA and HA-Fe is in the range of 1.2 V~5 mV, which shows that HA and HA-Fe materials are suitable for use as anode material. During the first change–discharge process of the HA-Fe material, additional irreversible charge plateaus (around 1.0 V) and discharge plateaus (around 1.5 V) exist, which may be due to the irreversible formation of the SEI film. The first discharge and charge capacities of HA-Fe material are 1038 mAh·g^−1^ and 437 mAh·g^−1^, respectively, corresponding to a low coulombic efficiency of 42%. Similar to many other anode materials, the irreversible capacity was also caused by the contribution of SEI formation results in the relatively low coulombic efficiency in the first charge/discharge process. The capacities of both HA and HA-Fe materials all increased with the change–discharge process. However, the increased degree of HA is not as large as that of HA-Fe. In the tenth cycle, the discharge capacity of HA-Fe is 146.9 mAh·g^−1^, while after 500 change–discharge cycles, the reversible capacity is 530 mAh·g^−1^, which is much higher than that of HA after 500 cycles (178 mAh·g^−1^).

To further explain the encouraging electrochemical properties of the synthesized HA-Fe materials, the electrochemical impedance spectroscopy (EIS) of HA and HA-Fe materials were conducted from 0.01 Hz to 100 kHz before cycling, after 100 cycles, and after 400 cycles, respectively. Nyquist plot analysis was performed using an equivalent circuit model, as shown in Figure S4a. The equivalent circuit model is made up of the following six electrical elements (R_s_, R_f_, R_ct_, CPE_1_, CPE_2_, and Q). The R_s_ corresponded to the ohmic resistance of the electrode system, while R_f_ and CPE_1_ corresponded to the resistance and capacitance attributed to the SEI film, respectively. CPE_2_ corresponded to the double-layer electrical capacitance. R_ct_ corresponded to the change–transfer resistance, and Q corresponded to the diffusion resistance in the solid phase, respectively [61]. This equivalent circuit model produced fitted lines as shown in Figure S4b–f, and the electrical element values are given in Table 2. From Table 2, it can be seen that before the cycling process, the resistance of HA (1403.6 Ω) was much larger than that of HA-Fe (389.6 Ω), which means that the addition of ferric ions can decrease the change–transfer resistance. After 100 cycles and 400 cycles of change–discharge processes, the change–transfer resistance of HA decreased to 240.7 Ω and 162.0 Ω, respectively, while the change–transfer resistance of HA-Fe decreased to 21.3 Ω and 13.7 Ω, respectively. The decreased change–transfer resistance during the change–discharge cycles can explain the obvious activation process of the HA-Fe material during the change–discharge process in Figure 4a.

Table 3 shows the electrochemical properties comparison between some previously reported HA or biomass macromolecular composites and the material reported in this work. It can be seen that the capacity of HA-Fe composites can rival most of the composites reported in the literature. Considering the renewability, low cost, the simple preparation process, and environment friendliness, HA-Fe has great commercial application potential.

### 3.3. Lithium Storage Mechanism Investigation

**Figure 5:**
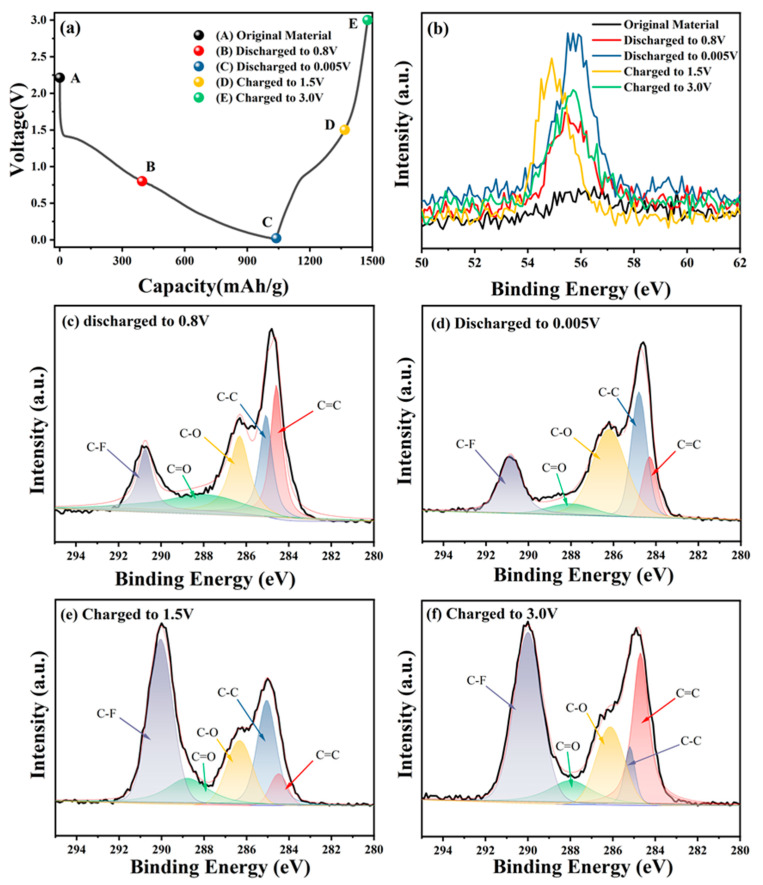
(**a**) The selected points for XPS measurements in the first discharge/charge cycle. (**b**) XPS profile of Li1s of the HA-Fe electrodes during the first discharge/charge cycle. (**c**,**d**) Ex-situ XPS spectra of C1s when discharged to 0.8 V, 0.005 V in the tenth discharge/charge cycle, respectively. (**e**,**f**) Ex-situ XPS spectra of C1s when recharged to 1.5 V, 3.0 V in the tenth discharge/charge cycle, respectively.

Ex-situ XPS was performed to characterize the electrodes at different electrochemical states in the first cycle to investigate the redox mechanism of the HA-Fe material during the change–discharge process, as shown in Figure 5a. The Li1s spectra of the HA-Fe electrode in different electrochemical states are shown in Figure 5b. In the case of the original electrode (A point), almost no Li1s signal could be detected in the XPS spectra. During the discharging process (A→B→C), the intensity of the Li1s peak increases continuously, which indicates that the continuous lithiation process is taking place in the HA-Fe. When the electrode is recharged (C→D→E), a gradual reduction of Li peak was detected by ex-situ X-ray photoelectron spectroscopy, indicating that the Li ions were gradually eliminated from the HA-Fe electrode. Nevertheless, when the electrode was recharged to 3.0 V, the Li1s peak could still be detected, indicating that not all lithium ions released from the HA-Fe material during the charge. The formation of SEI in the first change–discharge cycle and the irreversible reaction between HA-Fe and Li^+^ can explain this irreversible phenomenon.

As shown in Figure 5c–f, the peak intensity of the C1s peak during the change–discharge process was investigated in detail. The peaks at 284.05 eV, 284.5 eV, 286.31 eV, and 287.95 eV were assigned to the C=C, C-C, C-O, and C=O bonds, respectively. The intensity of the C=O peak dropped to its lowest value and the intensity of the C-O peak increased to its highest value when the voltage decreased from 0.8 V to 0.005 V, which illustrated that the C=O bond participated in the lithiation process and transferred to C-O bond. When recharged from 0.005 V to 1.5 V, the C-O peak decreased and the C=O peak increased, which illustrated the reversible redox transformation between the C=O double bond and the C-O single bond. However, as shown in Figure 5d,e, the intensities of C-C and C=C did not change obviously, which illustrated that the redox transfer from C-C to C=C nearly did not occur in the voltage range of 0.005 V–1.5 V. During the discharge process from 1.5 V to 3.0 V, the intensity of the C-C peak decreased and the C=C peak increased, which means that the C=C also participated in the lithiation process and transferred to the C-C bond.

Figure S5a–d showed the O1s characterization of the HA-Fe electrode at different states during the discharge–charge processes. As shown in Figure S5a,b, the C=O peak decreased during the discharge process from 0.8 V to 0.005 V, while the C-O peak increased accordingly, which is consistent with that from the C=O changing process in the C1s peak. In contrast, the C=O peak increased while the C-O peak decreased again during the charged process from 0.005 V to 1.5 V due to the reversible redox transformation between the C=O bond and the C-O bond (from Figure S5b,c). The obvious transformation process illustrated that the C=O is a very important active site for Li storage. In addition, it can be seen that the C=O bond nearly did not change from 1.5 V to 3.0 V, which illustrated that the C=O bond redox voltage range is 0.005 V~1.5 V but not 1.5 V~3.0 V. From the above discussion, it can be known that the voltage ranges of redox conversion between C-O and C=O are lower than the voltage ranges of redox conversion between C=C and C-C.

**Figure 6:**
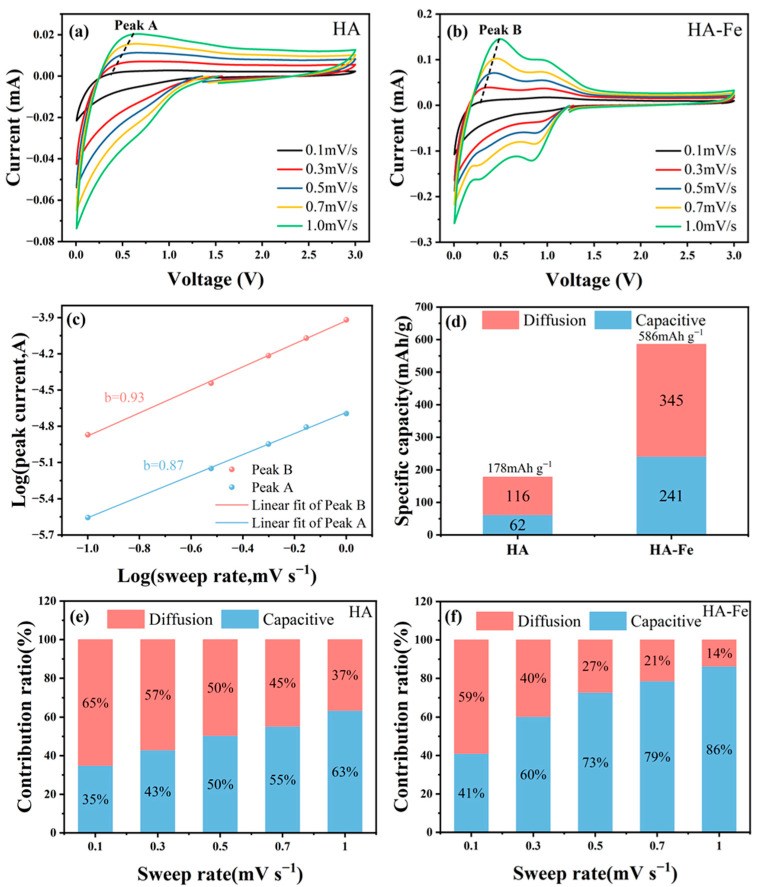
(**a**,**b**) CV curves at various scan rates ranging from 0.1 to 1.0 mV·s^−1^ of HA and HA-Fe. (**c**) Log(i) versus log (v) plots of HA and HA-Fe. (**d**) Total specific capacity with contributions from capacitive and diffusive processes of HA and HA-Fe at 0.1 mV·s^−1^. (**e**,**f**) Capacitive contribution at different scan rates of HA and HA-Fe, respectively.

The total stored charge comes from two processes: a diffusion controlled faradaic process and a capacitive process, which mainly includes a pseudocapacitance process that refers to the redox reaction taking place at the surface locations [66,67,68,69]. In order to study the lithium storage mechanism, the faradaic and capacitive contribution of HA and HA-Fe materials were calculated by CV curves at different scanning rates (0.1 mV·s^−1^, 0.3 mV·s^−1^, 0.5 mV·s^−1^, 0.7 mV·s^−1^, 1.0 mV·s^−1^), as shown in Figure 6a,b. The charge storage process can be characterized according to the equation: $\log\left(i\right)=log\left(a\right)\times b\;log(v)$. Where i represents the peak current in CV curve, v denotes the corresponding scan rate, and a and b are constants [66,70]. When the b value is close to 0.5, it indicates that the electrochemical process is controlled by the internal solid-phase diffusion process. And when the b value is close to 1, it indicates that the electrochemical process is controlled by the capacitive process [71]. As shown in Figure 6c, the b values of HA and HA-Fe were 0.87 and 0.93, respectively, which indicates that HA-Fe has more capacitive contribution than HA, which can illustrate that the surface area of the HA-Fe materials is larger than that of HA material. Furthermore, for a charge storage process combined with diffusion-controlled faradaic process and capacitive process, its current response i at a given potential is the sum of the above two contributions, which can be presented as: $i=k_{1}v+k_{2}v^{0.5}$ or $\frac{i}{v^{0.5}}=k_{1}v^{0.5}+k_{2}$. Where $k_{1}v$ and $k_{2}v^{0.5}$ correspond to the pseudocapacitive process and diffusion-controlled faradaic process, respectively [67,68,72]. Therefore, by obtaining $k_{1}$ and $k_{2}$ values at a constant potential, it is possible to determine the quantitative contributions of capacitive and diffusive processes at a given voltage. Figure 6d showed the total specific capacity with contributions from capacitive and diffusive processes of HA and HA-Fe at 0.1 mV·s^−1^. The HA-Fe material has both higher capacitive and diffusion capacities (241 mAh·g^−1^ and 345 mAh·g^−1^) than that of HA material (62 mAh·g^−1^ and 116 mAh·g^−1^), which means that the diffusion-controlled faradaic contribution capability and surface redox sites all increased after chelating with Fe ions. Figure 6e,f showed the capacitive contribution at different scan rates of the HA and HA-Fe, respectively. It can be seen that the capacitive contribution of HA-Fe is higher than that of HA at different scan rates, which is mainly due to the fact that the high specific surface area can provide a large number of active sites for the rapid embedding and de-embedding of lithium ions, thus improving the capacitance of the electrode.

## 4. Conclusions

In summary, a novel material, named HA-Fe, by chelating ferric ion with humic acid was prepared in this work and used as anode materials of LIBs. The HA-Fe materials were synthesized by a simple way without any heating or emission of toxic substances. According to the SEM, TEM, XPS, XRD, and nitrogen adsorption–desorption experimental results, it was found that the ferric ions can chelate with humic acid successfully under the mild conditions, and the ferric ions can increase the surface area of materials and transform HA into amorphous structure. HA-Fe has excellent electrochemical properties, including long cycle stability and excellent rate performance. A high specific capacity of 586 mAh·g^−1^ was maintained at 0.1 A·g^−1^ after 1000 cycles. In addition, when the current density increased from 0.05 A·g^−1^ to 1 A·g^−1^, the capacity of HA-Fe changed from 462 mAh·g^−1^ to 252 mAh·g^−1^ with the capacity retention rate of 55.63%, which is much higher than that of HA (32.55%) and Fe (24.85%). Moreover, the chelation between ferric ion and humic acid can improve the activation degree of HA-Fe material (improved about eight times) compared with pure HA material (improved about 2 times) during the change–discharge process. In the end, the intensified mechanism was also investigated with ex-situ XPS measurements. It was found that C=O and C=C bonds are activation sites for storing lithium ions, but with different redox voltages. In our opinion, the simple synthesis conditions and favorable electrochemical performance of this HA-Fe anodes make it a promising material for the development of truly powerful “green” LIBs.

## Figures and Tables

**Table 1:** Mass fractions of C, O, and Fe on the surface spectrum of HA and HA-Fe.

Mass Fractions of Different Element on Surface Spectrum (wt%)	C	O	Fe
HA	72.3	27.7	<0.1
HA-Fe	44.0	26.8	29.2

**Table 2:** Values of electrical elements in the equivalent circuit model.

Sample	Rs (Ω)			Rct (Ω)		
	Before Cycling	After 100 Cycles	After 400 Cycles	Before Cycling	After 100 Cycles	After 400 Cycles
HA	9.39	2.08	2.55	1403.6	240.7	162.0
HA-Fe	4.96	9.06	1.39	389.6	21.3	13.7

**Table 3:** Electrochemical properties of several HA or biomass macromolecular composites.

Sample	Current Density (A·g^−1^)	Capacity (mAh·g^−1^)	Cycles	Reference
HA-Fe	0.1	586	1000	This work
HA	0.04	180	200	[45]
H-CF	0.1	249	100	[62]
PTA-700	0.1	535	100	[63]
L-900	0.1C	433	100	[64]
PCS-CaCl2	0.2C	546	100	[65]

## Data Availability

The data are unavailable due to privacy.

## Supplementary Materials

The following supporting information can be downloaded at: https://www.mdpi.com/article/10.3390/ma16196477/s1, Figure S1. (a–d) SEM images with different magnifications of HA and HA-Fe. (e–g) SEM image of HA-Fe and the corresponding elemental mapping images of O (purple) and Fe (yellow). Figure S2. TGA curves of HA and HA-Fe. Figure S3. (a,b) Cyclic voltammetry measurements of HA and HA-Fe during the first three cycles in the voltage range of 0.005 V~3.0 V at 0.1 mV·s^−1^; (c) Cyclic voltammetry comparison plot of HA and HA-Fe at the 3rd cycle; (d) Cyclic voltammetry comparison plot of HA-Fe at the 3rd cycle and the 800th cycle. Figure S4. (a) The equivalent circuit model used to analyze the nyquist plots. (b,c) Electrochemical impedance plots of the HA-Fe, HA before cycling, after 100th cycling and after 400th cycling. (d–f) Electrochemical impedance comparison of HA and HA-Fe materials before cycling, after 100th cycling and after 400th cycling. Figure S5. (a) XPS spectra of O1s when discharged to 0.8 V, (b) O1s when discharged to 0.005 V, (c) O1s when charged to 1.5 V, (d) O1s when charged to 3.0 V.
