# Supplementary material for: FeIII Chelated with Humic Acid with Easy Synthesis Conditions and Good Performance as Anode Materials for Lithium-Ion Batteries
Source: Materials (Basel). 2023 Sep 29;16(19):6477. doi: 10.3390/ma16196477 (PMC10573477; doi:10.3390/ma16196477)
Supplement: Supplementary file 1 [file materials-16-06477-s001.zip › materials-2600664-supplementary.pdf]

materials

MDPI

Supplementary Materials

Fe^{III} Chelated with Humic Acid with Easy Synthesis Conditions and Good Performance as Anode Materials for Lithium-Ion Batteries

Hao Zhang [1], Youkui Wang [1], Ruili Zhao [1], Meimei Kou [1], Mengyao Guo [1], Ke Xu [1], Gang Tian [2], Xinting Wei [2], Song Jiang [1], Qing Yuan [1,3,*] and Jinsheng Zhao [1,3,*]

1 School of Chemistry and Chemical Engineering, Liaocheng University, Liaocheng 252059, China; zh1836958@163.com (H.Z.); m19861904240@163.com (Y.W.); zhao1784308@163.com (R.Z.); kmm2018705344@163.com (M.K.); gmy15275641513@163.com (M.G.); x15866598163@163.com (K.X.); jiangsong006@163.com (S.J.)
2 Shandong Tianyi New Energy Co., Ltd., Liaocheng 252059, China; tg1999@163.com (G.T.); 18863588007@139.com (X.W.)
3 Shandong Provincial Key Laboratory of Chemical Energy Storage and Novel Cell Technology, Liaocheng University, Liaocheng 252059, China
* Correspondence: yuanqing@lcu.edu.cn (Q.Y.); j.s.zhao@163.com (J.Z.)

Citation: Zhang, H.; Wang, Y.; Zhao, R.; Kou, M.; Guo, M.; Xu, K.; Tian, G.; Wei, X.; Jiang, S.; Yuan, Q.; et al. Fe^{III} Chelated with Humic Acid with Easy Synthesis Conditions and Good Performance as Anode Materials for Lithium-Ion Batteries. *Materials* **2023**, *16*, 6477. https://doi.org/10.3390/ma16196477

Academic Editor: Satyam Panchal

Received: 24 August 2023
Revised: 24 September 2023
Accepted: 27 September 2023
Published: 29 September 2023

Copyright: © 2023 by the authors. Licensee MDPI, Basel, Switzerland. This article is an open access article distributed under the terms and conditions of the Creative Commons Attribution (CC BY) license (https://creativecommons.org/licenses/by/4.0/).

Material characterization

The particle morphologies of the as-prepared powder were examined by a Hitachi Su-70 scanning electron microscopy (SEM, Hitachi Inc., Tokyo, Japan). The transmission electron microscopy (TEM, JEM-2100) was also taken to investigate the structural characterization of the materials. The infrared spectra were recorded on a NICOLET AVATAR 360 FTIR spectrometer with KBr pellets. X-ray photoelectron spectroscopy (XPS) was conducted with ESCALAB 250Xi spectrometer to determine the surface elemental compositions and their valence state of the as-prepared composites. The phase compositions of the composites are characterized using powder XRD in the 2θ range from 5 to 800 by using a Kigaka D/max 2500 X-ray diffractometer, advance diffractometer with Cu-Ka radiation, and a step scan mode was adopted with a scanning step of 0.02. The TGA measurement was carried out in N_2 flow at a heating rate of 5 °C per minute using Netzsch STA 449 F3 Jupiter44 analyzer. The porosity was determined by nitrogen adsorption and desorption at 77 K using an ASAP 2460-3 (Micromeritics) volumetric adsorption analyzer.

Electrochemical performance measurements

The electrochemical performances of HA and HA-Fe were tested using CR2032-type coin cells. Lithium foil and Celgard 2400 porous membrane were used as the counter electrode and separator, respectively. The electrolyte was 1 mol·L^{-1} $LiPF_6$ in a mixed solution of ethylene carbonate (EC), methyl ethyl carbonate (EMC), and dimethyl carbonate (DMC) (1:1:1, v/v). For anode testing, the electrode composition consists of 70 wt% active materials, 20 wt% acetylene black and 10 wt% PVDF. The current collector used in this work was copper foil, whose diameter is 12 mm. The electrode was dried at 100 °C for 24 hours to remove NMP before being pressed.

To test the electrochemical performance, the charge-discharge experiments were performed on a Land Battery Testing System with the voltage range between 0.005 V and 3.0 V versus Li/Li^+ at room temperature. The fabricated coin cells were also used for CV measurements with a scanning interval of 0.005 V-3 V (scan rate: 0.1 mV·s^{-1}) using an AutoLab PGSTAT302N electrochemical workstation (Switzerland Wantong). The cycling stability and rate capability were tested using a programmable computer-controlled battery charger (Land CT2001A, Wuhan, China). For comparison, the preparation process and electrochemical measurements for HA material were also proceeded as same as those of HA-Fe.

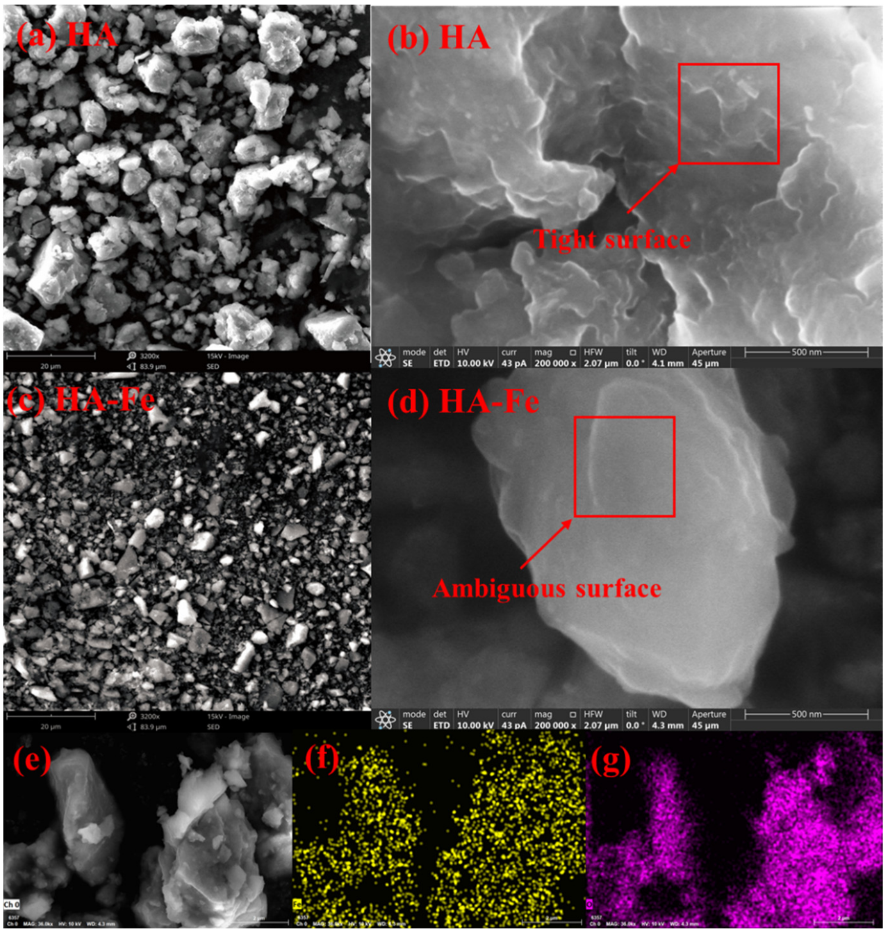

Figure S1. (a-d) SEM images with different magnifications of HA and HA-Fe. (e-g) SEM image of HA-Fe and the corresponding elemental mapping images of O (purple) and Fe (yellow).

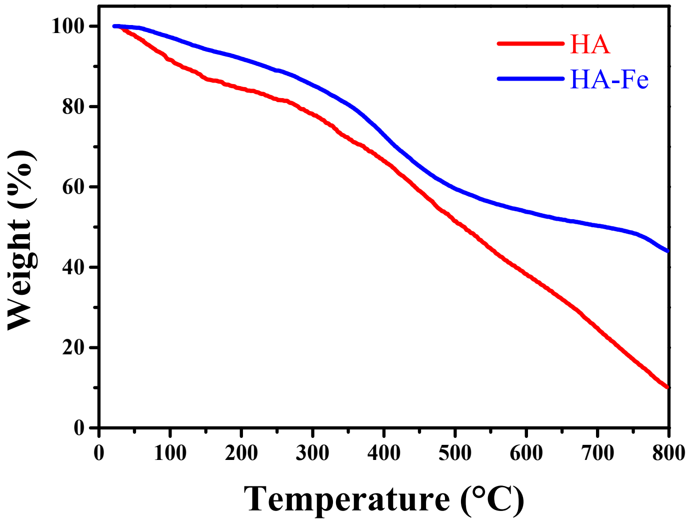

Figure S2. TGA curves of HA and HA-Fe.

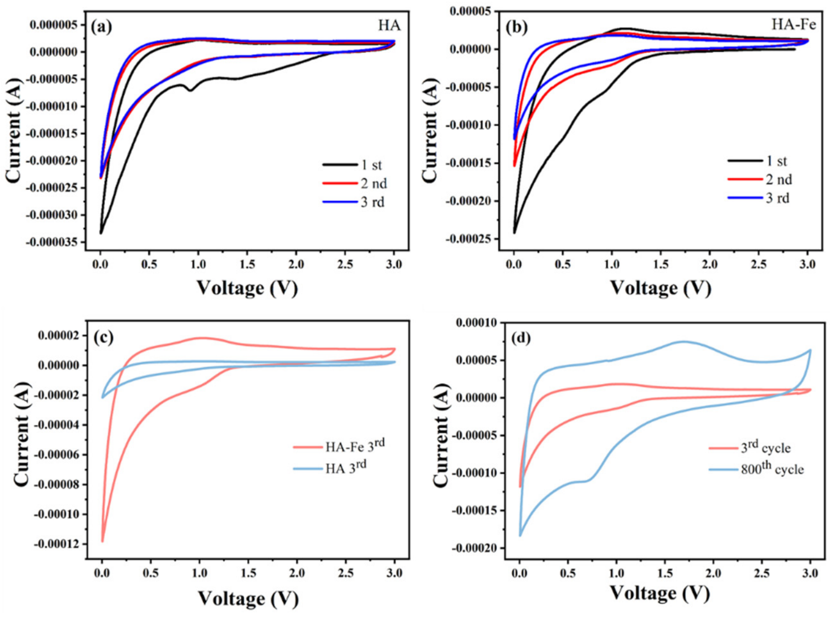

Figure S3. (a, b) Cyclic voltammetry measurements of HA and HA-Fe during the first three cycles in the voltage range of 0.005 V ~ 3.0 V at 0.1 mV·s^{-1}; (c) Cyclic voltammetry comparison plot of HA and HA-Fe at the 3rd cycle; (d) Cyclic voltammetry comparison plot of HA-Fe at the 3rd cycle and the 800th cycle.

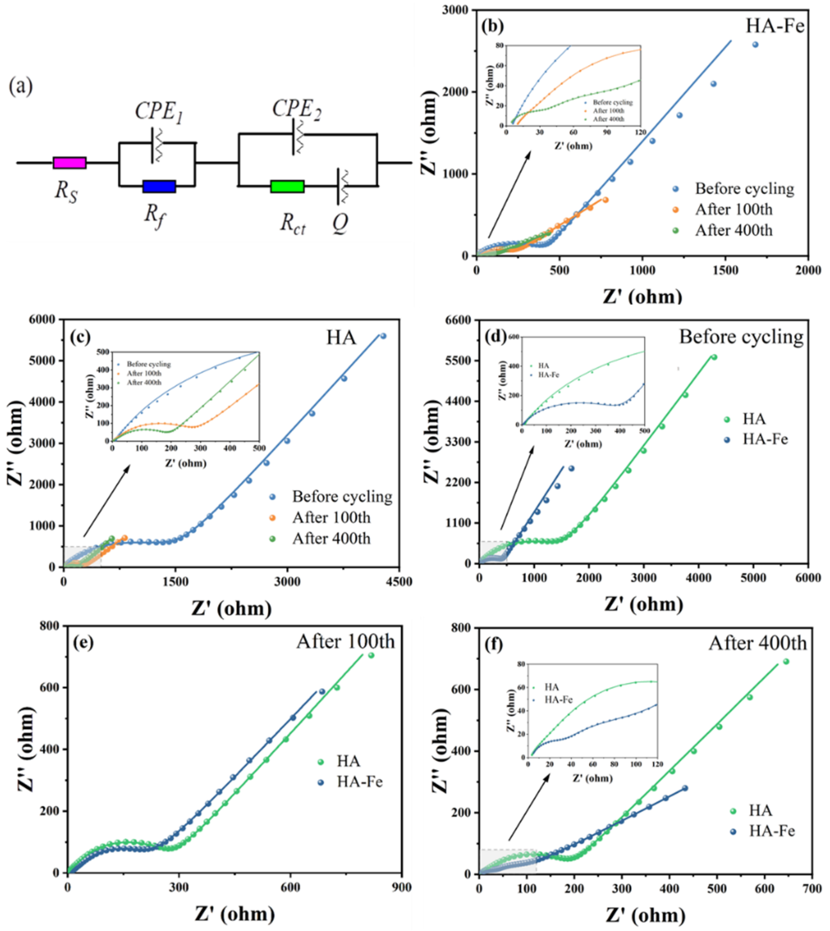

Figure S4. (a) The equivalent circuit model used to analyze the nyquist plots. (b, c) Electrochemical impedance plots of the HA-Fe, HA before cycling, after 100th cycling and after 400th cycling. (d-f) Electrochemical impedance comparison of HA and HA-Fe materials before cycling, after 100th cycling and after 400th cycling.

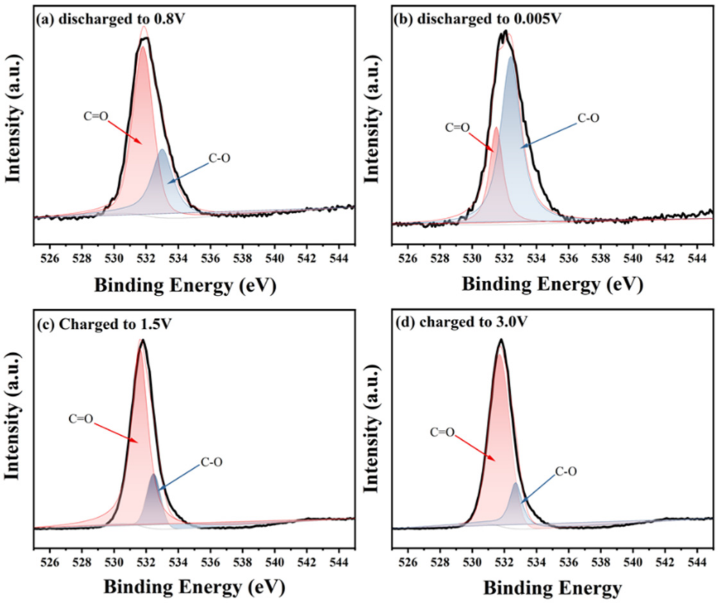

Figure S5. (a) XPS spectra of O1s when discharged to 0.8 V, (b) O1s when discharged to 0.005 V, (c) O1s when charged to 1.5 V, (d) O1s when charged to 3.0 V.
